# The Cardiovascular Effect of Systemic Homocysteine Is Associated with Oxidative Stress in the Rostral Ventrolateral Medulla

**DOI:** 10.1155/2017/3256325

**Published:** 2017-09-29

**Authors:** Mei-Fang Zhong, Yu-Hong Zhao, Hua Xu, Xing Tan, Yang-Kai Wang, Wei-Zhong Wang

**Affiliations:** ^1^Medical Training Center of Songjiang District, Shanghai 201620, China; ^2^Department of Medicine, Sijing Hospital, Shanghai 201601, China; ^3^Department of Nursing, Wuxi Higher Health Vocational Technology School, Wuxi 214028, China; ^4^Department of Physiology and Center of Polar Medical Research, Second Military Medical University, Shanghai 200433, China

## Abstract

It has been demonstrated that homocysteine (HCY) is a significant risk factor of hypertension, which is characterized by overactivity of sympathetic tone. Excessive oxidative stress in the rostral ventrolateral medulla (RVLM), a key region for control of sympathetic outflow, contributes to sympathetic hyperactivity in hypertension. Therefore, the goal of the present study is to determine the effect of systemic HCY on production of reactive oxygen species (ROS) in the RVLM. In the rat model of the diet-induced hyperhomocysteinemia (L-methionine, 1 g/kg/day, 8 weeks), we found that the HCY resulted in a significant increase (≈3.7-fold, *P* < 0.05) in ROS production in the RVLM, which was paralleled with enhanced sympathetic tone and blood pressure (BP). Compared to the vehicle group, levels of BP and basal renal sympathetic nerve activity in the HCY group were significantly (*P* < 0.05, *n* = 5) increased by an average of 27 mmHg and 31%, respectively. Furthermore, the rats treated with L-methionine (1 g/kg/day, 8 weeks) showed an upregulation of NADPHase (NOX4) protein expression and a downregulation of superoxide dismutase protein expression in the RVLM. The current data suggest that central oxidative stress induced by systemic HCY plays an important role in hypertension-associated sympathetic overactivity.

## 1. Introduction

Cardiovascular disease is a worldwide leading cause of morbidity and mortality in patients with heart failure, atherosclerosis, and hypertension. Individuals that suffered cardiovascular diseases usually possess an unhealthy food and dietary intake, which is closely associated with health-related conditions (e.g., hypertension) [1, 2]. It has been well estimated that homocysteine (HCY), derived from the dietary amino acid, is a causal agent for cardiovascular diseases [3]. In addition to being indisputably regarded as a biomarker of coronary artery disease and atherosclerosis changes [4, 5], hyperhomocysteinemia is also relative to the processing of demyelinization in the central nervous system (CNS) [6], and clinical studies suggest that the excess HCY level frequently parallels with neurodegenerative and acute disorders of CNS [7]. It has been widely established that the rostral ventrolateral medulla (RVLM), containing the sympathetic promoter neurons, is responsible for the central control of sympathetic vasomotor tone and blood pressure (BP) [8, 9]. Moreover, the elevated sympathetic tone is a pathophysiologic hallmark of hypertension and other cardiovascular dysfunctions [10]. There is accumulating evidences proving that oxidative stress plays an important role in the activation of the sympathetic nervous system and consequently hypertension [11]. Enhancement in oxidative stress in the RVLM contributes to the neural mechanisms of cardiovascular dysfunction in spontaneously hypertensive rats (SHRs) [12]. Oxidative stress mainly results from an imbalance between the production of reactive oxygen species (ROS), especially superoxide and the capacity of its scavenger such as superoxide dismutase (SOD). Activation of NADPH oxidase (NADPHase) is an important mechanism for ROS production and has been demonstrated to contribute to sympathetic overactivity in the RVLM of SHR [13, 14]. Thus, NADPHase and SOD are two crucial factors in maintaining the level of ROS production. It is reported that HCY increases ROS production in the intracellular levels, leading to atherosclerosis in vascular smooth muscle cells and neurotoxicity in neural stem cells [15, 16]. Coupled with oxidative stress, HCY is closely relative to the presence of hypertension [17]. However, the role of HCY in the activation of oxidative stress and sympathetic activity in the RVLM needs to be determined. Herein, we investigated the effects of systemic HCY on oxidative stress in the RVLM and further determined its underlying mechanism.

## 2. Materials and Methods

### 2.1. Animals and Experimental Treatments

Male Sprague-Dawley rats (12 weeks old) were purchased from Sino-British SIPPR/BK Laboratory Animal Ltd. (Shanghai, China) in the whole study. All of the procedures were guided and approved by the Animal Care and Use Committee of the Second Military Medical University and conducted specifically to the principles of the Institutional Animal Care.

Animals were divided into two groups. The HCY group at the age of 12 weeks was given by L-methionine (1 g/kg body weight per day) in tap water for a period of 8 weeks, as previously described [18]. The amount of water drank by animals every day was divided into two parts. The first part was mixed with L-methionine according to body weight of the individual, while the second one was the normal potable water without L-methionine. The control group was given equal volume of water. Eight weeks after HCY treatment, plasma HCY levels in rats were measured. Blood samples were taken from rats and were centrifuged for 10 min at 1500 rpm at 4°C, followed by collection of plasma for the evaluation of HCY. HCY reagent (Beijing Strong Biotechnologies Inc., Beijing, China) was used to detect the levels of HCY by an enzymatic cycling method with Beckman AU 5800 automatic biochemical analyzer (Beckman, USA).

### 2.2. Measurements of Cardiovascular Parameters

Using a noninvasive computerized tail-cuff system (ALC-NIBP; Shanghai Alcott Biotech Inc., Shanghai, China), as previously depicted [19], BP in conscious rats was measured at baseline (12 weeks of age) and then every 4 days until the end of the study period. After completion of 8-week L-methionine treatment, levels of BP, HR, and basal renal sympathetic nerve activity (RSNA) were measured in an anesthetized state (urethane 800 mg/kg and *α*-chloralose 40 mg/kg i.p.). Briefly, the right femoral artery was cannulated to collect the data of mean arterial pressure (MAP) and heart rate (HR) by the PowerLab system. The left renal sympathetic nerve was dissected retroperitoneally and put on a pair of silver recording electrodes to measure RSNA. The RSNA signal was amplified, filtered, integrated, sampled, and converted to a digital signal by the PowerLab system (AD Instruments). The basal RSNA was taken by the percent of the maximum value, as previously described [20]. Usually, the maximum (Max) RSNA was obtained 5 min after the rat was euthanized (pentobarbital sodium, 200 mg/kg). Background noise levels for RSNA were measured 15–20 min after the rat was euthanized. Using the unit conversion of the PowerLab Chart system, the Max was set to 100%, and the noise level was set to 0%. Baseline nerve activity was taken as the percent of the Max.

### 2.3. Detection of ROS Production in the RVLM

ROS production in the RVLM was detected by fluorescence, as previously delineated [21, 22]. Briefly, the rats were killed by an overdose of pentobarbital sodium (200 mg/kg). After being fixed and dehydrated in 4% paraformaldehyde and 20% sucrose, the brain of the rat was dissected into sections of 15 *μ*m and then incubated with dihydroethidium (DHE, 5 *μ*mol/L) at 37°C for 30 min. Brain sections were washed in cold PBS (0.1 M) 1 min for three times and finally examined using confocal laser scanning microscope. The excitation wavelength was 535 nm and the emission wavelength was 610 nm. The original images were acquired at red fluorescence microscope in the RVLM and were calculated by LAS-AF-Lite software.

### 2.4. Western Blot Analysis

Protein expression in the RVLM was detected by Western blot, as described previously [23]. After the RVLM tissues were punched from 100 *μ*m coronal sections of brainstem according to the rat atlas, they were immersed in cell lysate and centrifuged at 4°C for 20 min. The supernatant was left to determine the protein concentration and then applied to a 10% SDS-PAGE gel. Thereafter, transferring the protein sample to the PVDF membrane was performed and then, the membrane was blocked and incubated with NOX4 antibody (1 : 2000, Epitomics, America) or SOD1 antibody (1 : 2000, Epitomics, America) at 4°C overnight [23]. One day later, the membrane was combined with secondary antibody goat anti-rabbit IgG (H + L) for 2 h at room temperature, and the binds of protein were examined by the Syngene Bio Imaging system (Gene Company). Tubulin was severed as the loading control.

### 2.5. Statistical Analysis

Data are presented as mean ± SEM. The changes in BP by the tail-cuff system and the body weight between the vehicle and HHC groups were analyzed by two-way ANOVA with repeated measures, followed by Tukey' post hoc tests. The difference in the rest of this study between the two groups was calculated using unpaired *t*-test. The level of significance was set at *P* < 0.05 statistically.

## 3. Results

### 3.1. Establishment of the Diet-Induced HHC Rat Model

**Figure 1:**
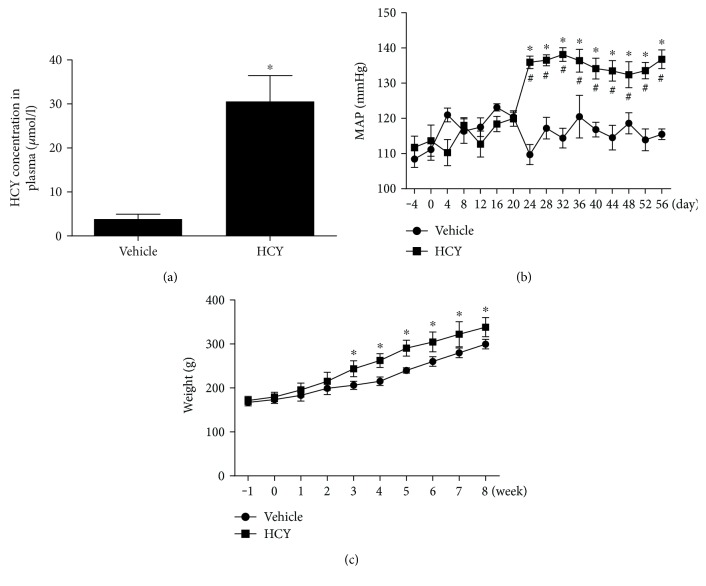
The concentration of HCY in plasma and changes in blood pressure and body weight in vehicle- and L-methionine-treated rats. Plasma HCY concentration (a), BP (b) obtained in conscious rats, and body weight (c) were significantly increased in the HCY group compared to the vehicle group. *n* = 5/group. ^∗^*P* < 0.05 versus the vehicle group, ^#^*P* < 0.05 versus 0 day.

As shown in Figure 1, the rat model of diet-induced hyperhomocysteinemia was identified by the assessment of plasma HCY concentration 8 weeks after L-methionine treatment (Figure 1(a)). It was found that a consistent elevation of mean arterial pressure (MAP) was induced by systemic treatment with L-methionine compared with vehicle treatment. In addition, the HCY rats showed a significance in body weight (Figure 1(b) and 1(c)). Compared with the control group, however, L-methionine treatment had no effect on heart rate.

### 3.2. Effects of HCY on BP, HR, and Basal RSNA Detected in Anesthetized Rats

**Figure 2:**
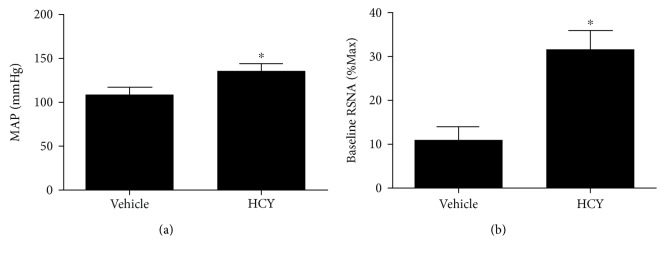
Levels of BP (a) and basal RSNA (b) obtained in anesthetized rats with L-methionine treatment. *n* = 5/group. ^∗^*P* < 0.05 versus the vehicle group.

Eight weeks after the L-methionine treatment, the rats were anesthetized to examine the value of BP, HR, and basal RSNA. Compared to the vehicle group, levels of MAP and basal RSNA in the HCY group were (*P* < 0.05, *n* = 5) increased by an average of 27 mmHg and 31%, respectively. There was no significance in HR between the HCY and control groups (Figure 2).

### 3.3. Detection of ROS Production in the RVLM

**Figure 3:**
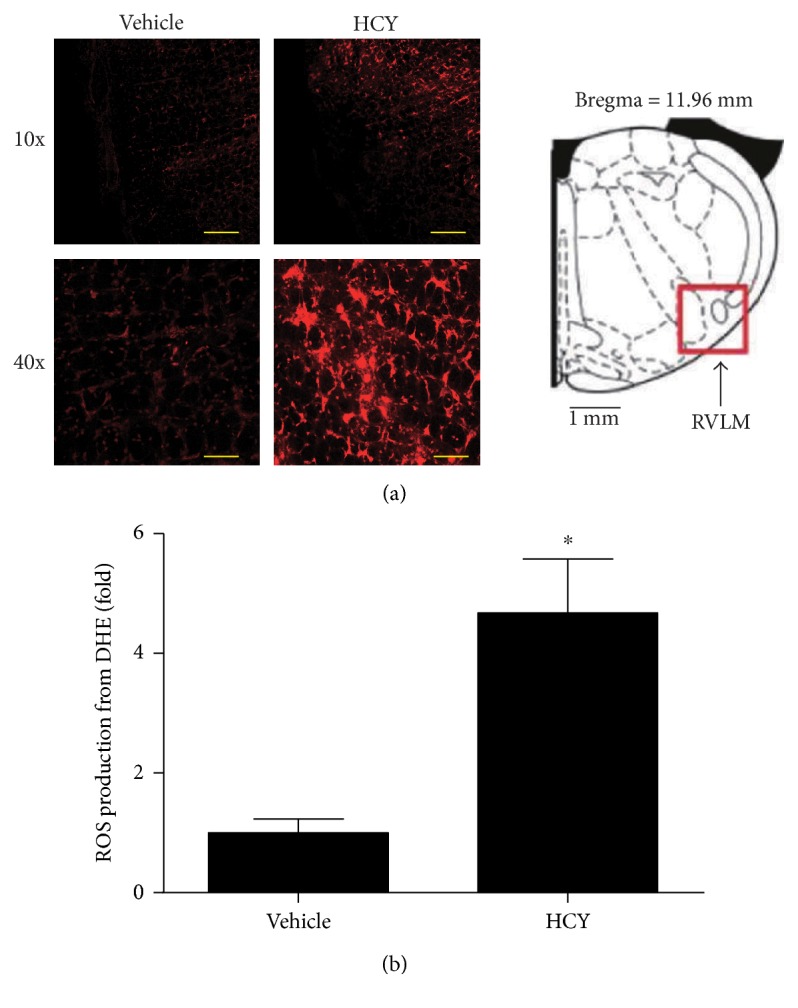
The ROS production in the RVLM in response to systemic HCY. (a) Representative images of ROS (red) by the DHE method in the RVLM region (right). Scale bars = 200 *μ*m in 10x magnification and 50 *μ*m in 40x magnification. (b) Quantification of ROS production in the RVLM. *n* = 5/group. ^∗^*P* < 0.05 versus the vehicle group.

In order to detect the change in ROS production in the RVLM by HCY, we performed the fluorescent labeling (DHE) to examine ROS production in the RVLM. As presented in Figure 3, L-methionine treatment caused a significant (*P* < 0.05, *n* = 5) increase in ROS production in the RVLM compared with vehicle treatment.

### 3.4. Protein Levels of NOX4 and SOD1 in the RVLM

**Figure 4:**
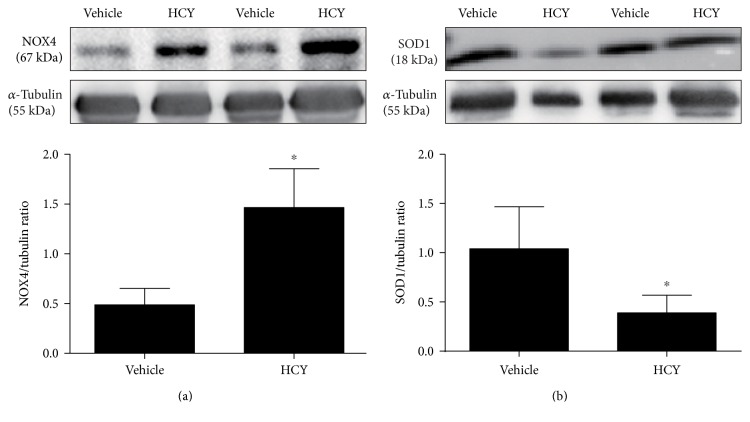
Protein levels of NOX4 and SOD1 in the RVLM in response to systemic HCY. As shown, NOX4 was dramatically increased within the HCY group (a) while SOD1 represented the opposite (b). *n* = 4‐5/group. ^∗^*P* < 0.05 versus the vehicle group.

As indicated in Figure 4, the protein expression of NOX4 in the RVLM was significantly (*P* < 0.05, *n* = 5) increased in rats treated L-methionine compared with vehicle. However, HCY significantly (*P* < 0.05, *n* = 5) reduced the expression of SOD1 protein.

## 4. Discussion

The main purpose of this study is to elucidate the effects of systemic HCY on ROS production in the RVLM. Our data shows that the diet-induced hyperhomocysteinemia leads to accelerated oxidative stress in the RVLM, which is associated with high levels in BP and sympathetic overactivity.

It is well known that the RVLM plays a key role in regulation of sympathetic out flow [24, 25]. The abnormalities of the RVLM in the regulation of sympathetic nerve activity contribute to cardiovascular dysfunction like chronic heart failure and hypertension [25, 26]. Previous studies have suggested that HCY plays a role as a neurotransmitter or neuromodulator in the medullary autonomic nuclei [27]. Therefore, our present study was designed to determine the relationship between hyperhomocysteinemia and increased sympathetic activity and BP at the level of the RVLM. It is suggested that hyperhomocysteinemia is an important contributor to high levels of sympathetic overactivity and BP in the RVLM. It has been demonstrated that there are multiple centers involved in cardiovascular regulation. The function of the other areas can also be influenced through systemic drug administration. We do not completely rule out the possibility that the effects of HCY on BP and the sympathetic nervous system are associated with the other areas. In this study, however, RVLM tissue was punched to analyze the changes in oxidative stress and confirmed that systemic HCY produced the oxidative stress at the level of the RVLM. So, oxidative stress at the level of the RVLM is at least involved in the central effect of HCY on BP.

Abundant evidences suggest that HCY acts as a risk factor for cardiovascular diseases such as chronic heart and renal failure, type II diabetes, and hypertension [4, 28, 29]. HCY, a sulfur amino acid which is synthesized from dietary methionine by a process of demethylation [30], has been demonstrated to be closely involved in the abnormal cardiovascular autonomic control and increased arterial pressure [31]. Increase in resting BP caused by hyperhomocysteinemia was totally reversed by interference with *β*1-adrenoceptor antagonist atenolol, indicating that the HCY-relative hypertension is associated with the increase in sympathetic activity [32]. It has been well established that ROS has a pivotal role in the activation of the central and peripheral sympathetic nerve system [12, 33]. Increased ROS production in the RVLM disrupts the balance of excitatory and inhibitory inputs by enhancing glutamatergic while attenuating GABAergic inputs to the RVLM, thereby leading to sympathoexcitation [34]. High salt intake has been reported to accelerate the progress of hypertension via oxidative stress in the RVLM [35]. Similarly, recent evidence shows that high fructose intake also results in hypertension by increasing ROS production in the RVLM via angiotensin II receptor 1 (AT1R) [36]. With respect to HCY mainly obtained from dietary amino conversion, accumulating evidence has demonstrated that HCY is able to positively trigger ROS production in rat vascular smooth muscle cells and aggravates ROS-induced impairment of transmitter release in neurodegenerative diseases [37, 38]. However, whether HCY is associated with deleterious ROS production in the RVLM remains unclear. In this study, we exactly authenticate that high HCY intake contributes to the augmented ROS production in the RVLM. Moreover, we find that the expression of NOX4 (a subtype of NADPHase) is significantly increased, whereas SOD1 was decreased in hyperhomocysteinemia rats compared to vehicle groups. ROS generated by NOX4 has been shown to initiate plenty of cardiovascular disorders such as myofibroblast and hypertension [39, 40]. Importantly, NOX4, a homolog of NOX2/gp91, has been confirmed to be expressed in the brain. Conversely, overexpression of the antioxidant SOD1 in microglia cells completely altered ROS production and the corresponding neurotoxic signaling [41]. On the basis of our study and previous findings, we put forward a hypothesis that hyperhomocysteinemia causes increased ROS production by modulating NOX4 and SOD1 activity in the RVLM.

Although HCY is highly associated with increased ROS production in the RVLM, the exact mechanisms by which HCY changes the expression of NADPHase and SOD are not further determined. HCY-induced imbalance of oxidative stress and redox state could be mediated by many intracellular signaling molecules, such as PI3K and P38/ERK [15]. In terms of this, we truly need to take further deep investigation to find a correlative signaling pathway to better illustrate the pro-ROS effect of HCY in the RVLM. Oxidative stress is often linked to inflammation; HCY is as well a proinflammatory factor that is able to stimulate C-reactive protein production in vascular smooth muscle cells, accelerating the pathogenesis of atherosclerosis [15, 37]. Interestingly, it is inclined to be a hotspot of the negative role of inflammation in the RVLM in the contribution to neurogenic hypertension [42, 43]. Nevertheless, whether HCY takes part in the progress of inflammation in the RVLM still remains unknown. In this study, a limitation was that we did not further investigate the effect of feeding folate on changes induced by HCY. It is reported that folic acid treatment reduces plasma HCY level and the angiotensin II-induced high blood pressure [44]. Furthermore, folic acid treatment is also capable of reducing oxidative stress in a rat model of pregnancy-induced hypertension [45]. Therefore, it is possible that feeding folate to rats reverses oxidative stress in the RVLM induced by systemic HCY. These questions need to be investigated in our future study.

In conclusion, the present study suggests that high HCY diet-induced hyperhomocysteinemia acts as a risk factor for oxidative stress in the RVLM, leading to sympathoexcitation and hypertension. It consolidates the theories of treating neurogenic hypertension by targeting HCY and reinforces the importance of taking a healthy diet.
